# The Role of Lipids Mediators in Inflammation and Resolution

**DOI:** 10.1155/2015/605959

**Published:** 2015-03-24

**Authors:** Alexandre de Paula Rogerio, Carlos Artério Sorgi, Ruxana Sadikot, Troy Carlo

**Affiliations:** ^1^Departamento de Clínica Médica, Laboratorio de Imunofarmacologia Experimental (LIFE), Instituto de Ciências da Saúde, Universidade Federal do Triangulo Mineiro (UFTM), Rua Vigário Carlos 162, 38025-350 Uberaba, MG, Brazil; ^2^Faculdade de Ciencias Farmacêuticas de Ribeirão Preto, Universidade de São Paulo, 14049-903 São Paulo, SP, Brazil; ^3^Pulmonary, Critical Care, Sleep and Allergy Medicine, University of Florida, Gainesville, FL 32610, USA; ^4^Pulmonary and Critical Care Medicine Division, Brigham and Women's Hospital, Boston, MA 02115, USA

Acute inflammation is generally self-limited. However, if acute inflammation fails to resolve, chronic inflammation can persist. The innate and adaptive immune systems, as well as structural cells, modulate the length and intensity of inflammatory responses. Aberrant immune responses, including those induced by allergens, environmental pollutants, infectious agents, acids, and other noxious stimuli, promote excessive leukocyte recruitment and the production of proinflammatory cytokines, lipids mediators, and chemokines, which are critical to initiate and maintain the inflammatory process. Lipids mediators, derived from the omega-6 polyunsaturated fatty acids (PUFA) including leukotrienes (LTs) and prostaglandins (PGs), are potent enhancers of innate and adaptive immune activity and are implicated in numerous inflammatory disorders. Yet certain PGs, such as PGD_2_ and PGE_2_, demonstrate anti-inflammatory effects. Similarly, lipoxins (LXs), derived from the omega-6 PUFA arachidonic acid, not only harbor potent anti-inflammatory activity, but also promote the resolution of inflammation. Complete resolution of inflammatory responses is critical for human health. Resolution is an active process that is regulated, in part, by specialized proresolving mediators such as the omega-3 PUFA derived resolvins, maresins, and protectins, in addition to the aforementioned LXs. These biochemical mediators signal through distinct receptors to both dampen inflammation and promote resolution.

This special issue covers the most recent research elucidating the role of these lipids mediators in inflammation and the resolution of inflammation.

In recent decades, considerable progress has been made in understanding the role of lipoxin A_4_ in health and disease. Two elegant reviews by Higgins et al. and Martini et al. illustrate the critical role LXA_4_ plays in patients with cystic fibrosis and neurological diseases, respectively.

Cystic fibrosis, an autosomal disease, leads to, among others, devastating infection and inflammation of the airways. Patients suffering from cystic fibrosis display decreased LXA_4_ production when compared to healthy individuals suggesting that this decrement contributes to continuous local inflammation. Interestingly, LXA_4_ triggers responses in bronchial epithelial cells that would be beneficial to CF patients. Exposure to LXA_4_ stimulates a rapid and transient intracellular Ca^2+^ increase and whole-cell Cl^−^ currents that restore fluid transport in cystic fibrosis, increases the airway surface liquid height via P2Y11 activation, and enhances epithelial cell migration and proliferation, activities crucial to the repair of epithelia. Thus, LXA_4_ demonstrates therapeutic potential for patients with cystic fibrosis.

Neurological diseases and conditions, such as Alzheimer's, Parkinson's, traumatic brain injury, and stroke as well as conditions leading to chronic neuropathic pain, typically present marked transient or continued neuroinflammation. Interestingly, Alzheimer's patients are slow to resolve inflammation and display lower LXA_4_ levels in cerebrospinal fluid and hippocampus samples compared to control subjects. Aspirin-triggered 15-epi-lipoxin A_4_ promoted decreased inflammation in a murine model of Alzheimer's by reducing proinflammatory and increasing anti-inflammatory mediators in the brain. Taken together, these demonstrate the neuroprotective properties of LXA_4_.

Protozoan infections cause serious health, political, social, and economic problems. In an experimental model, Sacramento et al. demonstrated that 5-lipoxygenase knockout animals displayed increased susceptibility to infection with* Leishmania infantum* as measured by an increase in parasitic load in several organs as well as decreased neutrophil migration to the infectious foci. In addition to these effects, reductions in proinflammatory cytokines involved in T cell differentiation to Th17 axis were observed. These results demonstrated that LTs play an important role in the controlling of* L. infantum*-induced visceral leishmaniasis.

Cysteinyl leukotrienes (cysLTs), like LTs, play an important role in diseases, such as asthma. Allergic asthma is a complex inflammatory disorder characterized by airway hyperresponsiveness, eosinophilic inflammation, hypersecretion of mucus, and tissue remodeling. The asthma pathophysiology involves chemical mediators that play an important role in the establishment of inflammation. Baptista-dos-Reis et al. review the roles of cysLTs in eliciting eosinophil granule protein secretion and emphasize the importance of this finding in eosinophil immunobiology and in eosinophilic diseases.

Allergen exposure may induce changes in brainstem secondary neurons, with neural sensitization of the nucleus solitary tract, which can be considered one of the causes of the airway hyperresponsiveness, a characteristic feature of asthma. Based on these considerations, Spaziano et al. evaluated functional, morphological, and biochemical changes occurring in the nucleus solitary tract following airway sensory nerve activation in naive and ovalbumin sensitized rats.

The role of inflammation in diabetes is widely known. Tessaro et al. carefully review the roles eicosanoids play in diabetes-related nephropathy, retinopathy, and cardiovascular events.

